# Viral Factors Important for Efficient Replication of Influenza A Viruses in Cells of the Central Nervous System

**DOI:** 10.1128/JVI.02273-18

**Published:** 2019-05-15

**Authors:** Jurre Y. Siegers, Marco W. G. van de Bildt, Zhanmin Lin, Lonneke M. Leijten, Rémon A. M. Lavrijssen, Theo Bestebroer, Monique I. J. Spronken, Chris I. De Zeeuw, Zhenyu Gao, Eefje J. A. Schrauwen, Thijs Kuiken, Debby van Riel

**Affiliations:** a Department of Viroscience, Erasmus MC Rotterdam, Rotterdam, the Netherlands; b Department of Neuroscience, Erasmus MC Rotterdam, Rotterdam, the Netherlands; St. Jude Children's Research Hospital

**Keywords:** CNS disease, H1N1, H3N2, H5N1, influenza A virus, encephalitis, extrarespiratory, pathogenesis, viral replication, virus attachment

## Abstract

Central nervous system (CNS) disease is one of the most common extrarespiratory tract complications of influenza A virus infections. Remarkably, zoonotic H5N1 virus infections are more frequently associated with CNS disease than seasonal or pandemic influenza viruses. Little is known about the interaction between influenza A viruses and cells of the CNS; therefore, it is currently unknown which viral factors are important for efficient replication. Here, we determined the replication kinetics of a seasonal, pandemic, zoonotic, and lab-adapted influenza A virus in human neuron-like (SK-N-SH) and astrocyte-like (U87-MG) cells and primary mouse cortex neurons. In general, highly pathogenic avian influenza (HPAI) H5N1 virus replicated most efficiently in all cells, which was associated with efficient attachment and infection. Seasonal H3N2 and to a lesser extent pandemic H1N1 virus replicated in a trypsin-dependent manner in SK-N-SH but not in U87-MG cells. In the absence of trypsin, only HPAI H5N1 and WSN viruses replicated. Removal of the multibasic cleavage site (MBCS) from HPAI H5N1 virus attenuated, but did not abrogate, replication. Taken together, our results showed that the MBCS and, to a lesser extent, the ability to attach are important determinants for efficient replication of HPAI H5N1 virus in cells of the CNS. This suggests that both an alternative hemagglutinin (HA) cleavage mechanism and preference for α-2,3-linked sialic acids allowing efficient attachment contribute to the ability of influenza A viruses to replicate efficiently in cells of the CNS. This study further improves our knowledge on potential viral factors important for the neurotropic potential of influenza A viruses.

**IMPORTANCE** Central nervous system (CNS) disease is one of the most common extrarespiratory tract complications of influenza A virus infections, and the frequency and severity differ between seasonal, pandemic, and zoonotic influenza viruses. However, little is known about the interaction of these viruses with cells of the CNS. Differences among seasonal, pandemic, and zoonotic influenza viruses in replication efficacy in CNS cells, *in vitro*, suggest that the presence of an alternative HA cleavage mechanism and ability to attach are important viral factors. Identifying these viral factors and detailed knowledge of the interaction between influenza virus and CNS cells are important to prevent and treat this potentially lethal CNS disease.

## Abstract (precis)

Central nervous system (CNS) disease is one of the most common extrarespiratory tract complications of influenza A virus infections, and the frequency and severity differ between seasonal, pandemic, and zoonotic influenza viruses. However, little is known about the interaction of these viruses with cells of the CNS. Differences among seasonal, pandemic, and zoonotic influenza viruses in replication efficacy in CNS cells, *in vitro*, suggest that the presence of an alternative HA cleavage mechanism and ability to attach are important viral factors. Identifying these viral factors and detailed knowledge of the interaction between influenza virus and CNS cells are important to prevent and treat this potentially lethal CNS disease.

## INTRODUCTION

One of the most common extrarespiratory complications of influenza virus infection is central nervous system (CNS) disease (1, 2). Clinically, CNS disease can range from mild febrile seizures to severe or even fatal meningoencephalitis (2, 3). Although most studies on influenza virus-associated CNS disease have focused on influenza A viruses, viruses of type B are also able to cause CNS disease. This is, however, less frequently observed (2, 4). Influenza A viruses, hereinafter referred to as influenza virus, have been linked to CNS disease since the 1918 H1N1 pandemic (5, 6), and CNS disease has been observed during all subsequent pandemics (7–12) as well as during seasonal epidemics, with sporadic detection of influenza virus in the CNS or cerebral spinal fluid (CSF) of humans (13–15). Zoonotic influenza viruses only occasionally infect humans, but when they do, they are frequently associated with severe and systemic disease (1). Highly pathogenic avian influenza (HPAI) H5N1 and H7N9 viruses, two recent zoonotic influenza viruses, are both associated with CNS disease (16–19). The HPAI H5N1 virus is possibly the most neurotropic influenza virus known and has frequently been associated with CNS disease in humans and in other naturally (20–23) and experimentally (24–27) infected mammalian species.

In order to infect, replicate in, and spread throughout the CNS, influenza viruses first have to be able to enter the CNS. Entry of influenza viruses into the CNS can occur via, for example, the olfactory (24, 26–28), trigeminal (6, 27, 29, 30), vagus (29–31), and sympathetic (27, 31) nerves and possibly other cranial nerves. The primary targets of influenza viruses are, however, epithelial cells of the respiratory tract (32), which differ from cells of the CNS. Influenza virus infection starts with attachment of the virus to sialic acids (SA) present on host cells (33). Human and avian influenza viruses attach preferentially to α-2,6- and α-2,3-linked SA, respectively, present in the upper and lower respiratory tracts of humans, respectively (33). In cells of the CNS, little is known about SA distribution on the different cells at different anatomical locations. One comparative study using lectin immunohistochemistry suggested that in humans, both α-2,6 and α-2,3 SA are present on neurons and glial cells in many different regions, including cerebral cortex, hippocampus, brainstem, and cerebellum (34). In the mouse brain, however, SA distribution is less widespread, and regions with and without detectable SA are infected with influenza viruses (34). In another study, it was found that in human cortex tissue, some neurons only express α-2,3 SA, oligodendrocytes mainly express α-2,6 SA, while astrocytes appear to express both receptors (35). Moreover, both α-2,3 and α-2,6 SA receptors have been found to be present on human neuroblastoma SK-N-SH and SH-SY5Y and human glioblastoma T98G cell lines (36, 37). Given these differential results as well as the fact that SA usage depends on more than α-2,3 and α-2,6 SA linkage, e.g., α-2,8 SA linkage (38, 39) or even SA-independent entry of the virus (40), more studies should reveal which viruses are able to attach to cells in the CNS.

In order for progeny viruses to infect new cells, cleavage of the immature surface protein hemagglutinin (HA) into the biologically activated and infectious form is required (41). Influenza viruses that contain a monobasic cleavage site can be cleaved by trypsin-like serine proteases such as human airway trypsin-like protease (HAT), transmembrane serine protease 2 (TMPRSS2), TMPRSS4, or matriptase present in the human respiratory tract (42–44). In the human CNS, expression of HAT in the cerebellum (45) and matriptase mRNA in the frontal and temporal cortices, hippocampus, and cerebellum have been reported (46). Viruses that contain a multibasic cleavage site (MBCS), such as the HPAI H5N1 virus, can be cleaved by ubiquitously expressed subtilisin-like proteases such as furin and PC5/6 (41, 47). This MBCS is an important factor contributing to the ability to spread systemically, including in the CNS. Although extrarespiratory spread of HPAI H5N1 virus depends on the presence of the MBCS in ferrets, insertion of an MBCS into a seasonal H3N2 virus did not result in efficient systemic replication in ferrets, suggesting that more factors are necessary (24, 48). Other viruses that are associated with CNS invasion in mice or ferrets are 1918 H1N1 and A/WSN/33 viruses (6, 49, 50). These viruses do not possess an MBCS but use a different protease-mediated mechanism for HA cleavage, allowing trypsin-independent replication. Taken together, virus receptor specificity, receptor availability on host cells, protease distribution and availability, and HA cleavage mechanism all seem to play important roles in influenza virus infection and cell tropism as well as replication efficiency in the respiratory tract and beyond.

To date, not much is known about the replication efficiency of different influenza viruses, especially seasonal viruses, in cells of the CNS. Thus far, evidence from both *in vivo* and *in vitro* studies suggests that HPAI H5N1 viruses are able to infect and replicate in neurons and astrocytes (17, 20–27, 36, 51–54), but a direct comparison of replication efficiency in cells of the CNS between seasonal, zoonotic, and pandemic influenza viruses is currently lacking. Similarly, insights into the roles of attachment, protease availability, and presence of an MBCS on replication efficiency in cells of the CNS for these viruses are lacking. Therefore, we here determined the virus attachment, infectivity, and replication kinetics of a seasonal H3N2, 2009 pandemic H1N1 (pH1N1), HPAI H5N1, and WSN viruses in human neuroblastoma (SK-N-SH), human astrocytoma (U87-MG), primary mouse cortex neurons (pmCortex), and Madin-Darby canine kidney (MDCK) cells. Subsequently, we established the importance of the MBCS for the replication efficiency of HPAI H5N1 virus in cells of the CNS.

## RESULTS

### HPAI H5N1 virus replicates more efficiently in CNS cells than H3N2 and pH1N1 viruses.

**FIG 1:**
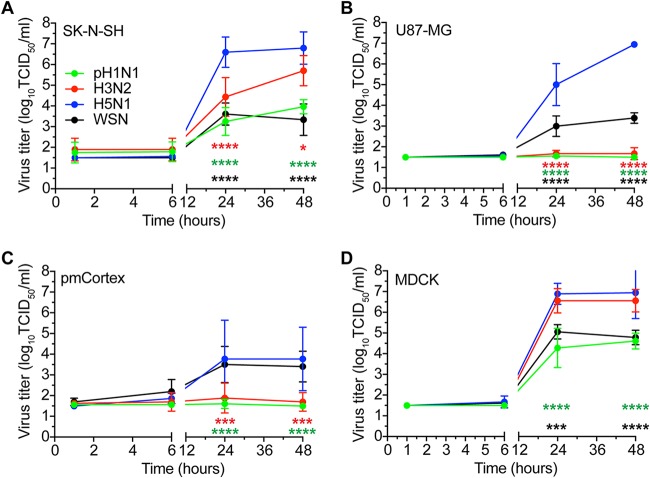
In the presence of trypsin, HPAI H5N1 virus replicates most efficiently in SK-N-SH, U87-MG, pmCortex, and MDCK cells. (A to D) Growth kinetics of pH1N1, H3N2, HPAI H5N1, and WSN viruses in SK-N-SH, U87-MG, pmCortex, and MDCK cells (MOI of 0.1) in the presence of trypsin. Data are presented as means ± SDs from at least three independent experiments. Two-way analysis of variance (ANOVA) with Dunnett’s multiple-comparison tests for individual viruses against HPAI H5N1 virus. *, *P* ≤ 0.05; ***, *P* ≤ 0.001; ****, *P* ≤ 0.0001.

The replication kinetics of pH1N1, H3N2, HPAI H5N1, and WSN viruses were determined in SK-N-SH, U87-MG, pmCortex, and MDCK cells in the presence of trypsin. All viruses replicated efficiently in MDCK cells, where HPAI H5N1 virus and H3N2 virus replicated to higher titers than pH1N1 virus and WSN virus (Fig. 1D). In both SK-N-SH and U87-MG cells, HPAI H5N1 virus replicated to a significantly higher titer (∼7 log_10_ 50% tissue culture infective dose [TCID_50_]/ml) than all other viruses (Fig. 1A and B). In U87-MG and pmCortex cells, only HPAI H5N1 and WSN viruses were able to replicate (Fig. 1B and C). In addition to HPAI H5N1 virus, SK-N-SH cells supported replication of pH1N1, H3N2, and WSN viruses, reaching virus titers of ∼5.7, ∼3.9, and ∼3.3, respectively (Fig. 1A). Overall, our results show that HPAI H5N1 and WSN viruses replicated in all cells investigated and that H3N2 and pH1N1 viruses replicated less efficiently in SK-N-SH cells and not at all in U87-MG cells and pmCortex cells.

### HPAI H5N1 and WSN viruses infected cells more efficiently than H3N2 and pH1N1 viruses.

**FIG 2:**
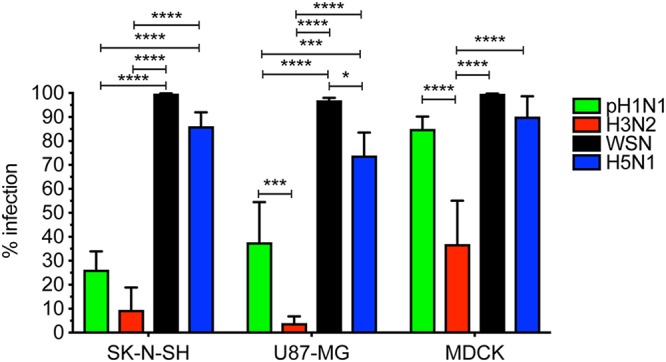
HPAI H5N1 and WSN viruses infect cells more efficiently than pH1N1 and H3N2 viruses. Percentages of infection in SK-N-SH, U87-MG, and MDCK cells were determined by FACS 8 hpi with either pH1N1, H3N2, WSN, or HPAI H5N1 virus (MOI of 3). Data are presented as means ± SDs from at least three independent experiments. Statistical analysis was performed using the two-way ANOVA with Tukey’s multiple-comparison test. *, *P* ≤ 0.05; ***, *P* ≤ 0.001; ****, *P* ≤ 0.0001.

To determine whether efficient replication was associated with the ability of the virus to enter and infect host cells, we determined the percentage of infection 8 h postinfection (hpi) (multiplicity of infection [MOI] of 3) in SK-N-SH, U87-MG, and MDCK cells, measured by flow cytometry (Fig. 2). In MDCK cells, pH1N1, HPAI H5N1, and WSN viruses infected significantly more cells than H3N2 virus. In SK-N-SH and U87-MG cells, HPAI H5N1 and WSN viruses infected significantly more cells than H3N2 or pH1N1 viruses.

### pH1N1 and H3N2 viruses attach less efficiently to SK-N-SH and U87-MG cells than HPAI H5N1 and WSN viruses.

**FIG 3:**
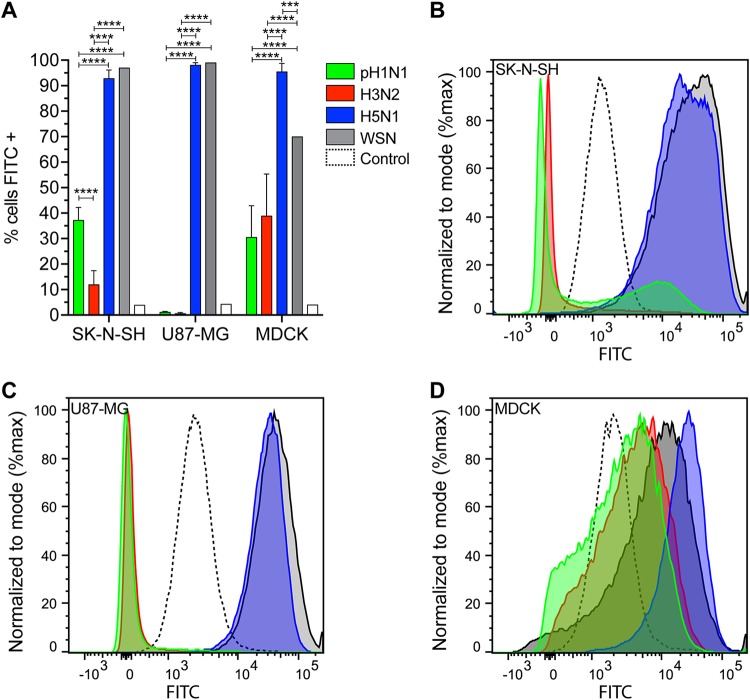
HPAI H5N1 and WSN viruses attach more efficiently to SK-N-SH and U87-MG cells than pH1N1 and H3N2 viruses. Virus attachment of pH1N1, H3N2, HPAI H5N1, and WSN viruses (using 100 hemagglutination units [HAU] units) to SK-N-SH, U87-MG, and MDCK cells. (A) Percentages of cells to which viruses attached. (B to D) Representative histograms of SK-N-SH, U87-MG, and MDCK cells. Dotted lines indicate cell control. Data in panel A are presented as means ± SDs from at least three independent experiments. Two-way ANOVA with Tukey’s multiple-comparison test. ***, *P* ≤ 0.001; ****, *P* ≤ 0.0001.

To determine whether there were differences in attachment between the viruses and whether this was associated with the infection percentages, we performed a virus attachment assay. The attachment efficiency was scored as follows: inefficient attachment (0% to 5%), low attachment (6% to 25%), intermediate attachment (26% to 75%), and efficient attachment (>76%). HPAI H5N1 virus attached efficiently (>90%) to all cell lines investigated (Fig. 3). WSN virus attached efficiently (>95%) to SK-N-SH and U87-MG cells and intermediately (70%) to MDCK cells (Fig. 3). Pandemic H1N1 virus attached with intermediate efficiency to SK-N-SH (37%) and MDCK (30%) cells (Fig. 3A, B, and D). Seasonal H3N2 virus attached with low efficiency to SK-N-SH (12%) and intermediate efficiency to MDCK (39%) cells (Fig. 3A, B, and D). Neither pH1N1 nor H3N2 virus attached to U87-MG cells (<2%) (Fig. 3A and C). Overall, these results show that pH1N1 and H3N2 viruses attach less efficiently to SK-N-SH and U87-MG cells than H5N1 and WSN viruses.

### HPAI H5N1 and WSN viruses replicate in the absence of trypsin, but H3N2 and pH1N1 viruses do not.

**FIG 4:**
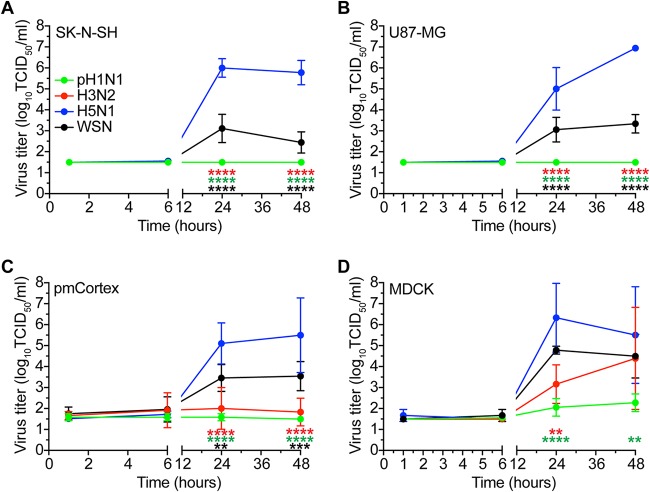
In the absence of trypsin, HPAI H5N1 virus grows most efficiently in SK-N-SH, U87-MG, MDCK, and pmCortex cells. (A to D) Growth kinetics of pH1N1, H3N2, HPAI H5N1, and WSN viruses in SK-N-SH, U87-MG, MDCK, and pmCortex cells (MOI of 0.1) in the absence of trypsin. Data are presented as means ± SDs from at least three independent experiments. Two-way ANOVA with Dunnett’s multiple-comparison test against H5N1 virus. **, *P* ≤ 0.01 ***, *P* ≤ 0.001; ****, *P* ≤ 0.0001.

**FIG 5:**
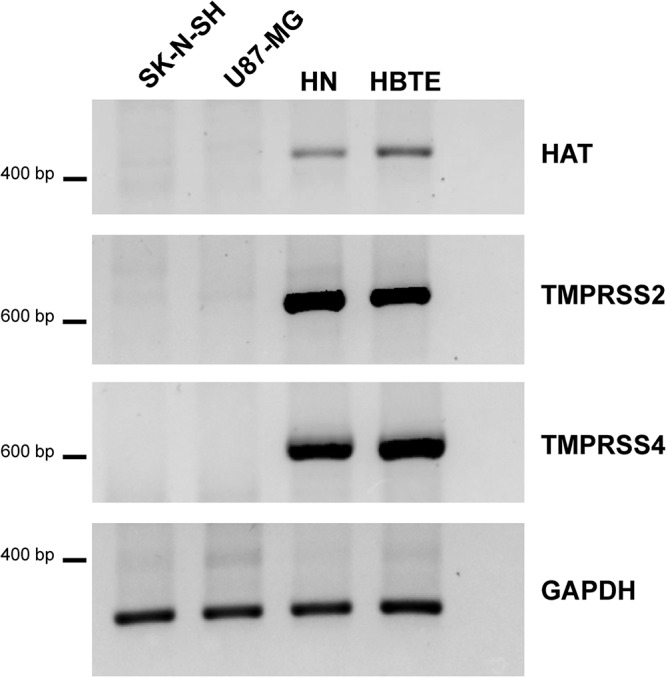
HAT, TMPRSS2, and TMPRSS4 mRNAs are not present in SK-N-SH and U87-MG cells. Presence of three known HA cleaving enzymes: human airway trypsin (HAT), transmembrane serine protease 2 (TMPRSS2), and TMPRSS4. Positive-control cell lines human nasal cells (HN) and human bronchial/tracheal epithelial (HBTE) cells did express HAT, TMPRSS2, and TMPRSS4 mRNA.

To test whether efficient replication is dependent on the presence of trypsin, we determined the replication kinetics in the absence of trypsin. Replication of HPAI H5N1 and WSN viruses was not affected by the absence of trypsin (Fig. 4). In the presence of trypsin, H3N2 and pH1N1 viruses replicated efficiently in SK-N-SH cells but not in the absence of trypsin (Fig. 4A). To further understand this finding, we analyzed the presence of specific host cell proteases known to cleave the HA protein of H3N2 and pH1N1 viruses (43). We found that neither SK-N-SH nor U87-MG cells expressed HAT, TMPRSS2, nor TMPRSS4 mRNA, whereas these transcripts were present in human nasal cell (HN) cultures and human bronchial/tracheal epithelial (HBTE) cultures (Fig. 5). These results show that HPAI H5N1 and WSN viruses replicate independently of trypsin and that pH1N1 and H3N2 viruses are dependent on trypsin for replication in SK-N-SH cells.

### The MBCS of HPAI H5N1 virus is important but not solely responsible for replication in SK-N-SH cells.

**FIG 6:**
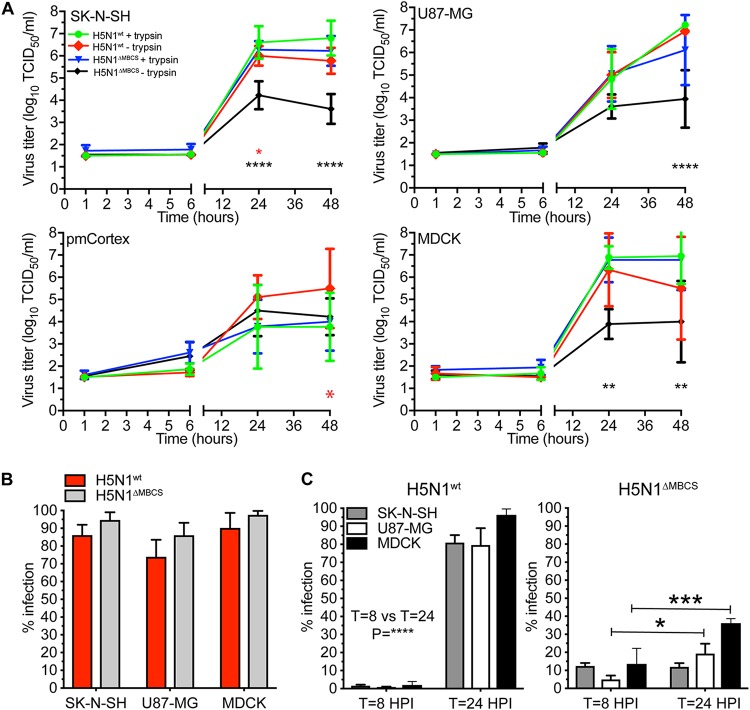
H5N1 virus without an MBCS replicates less efficiently in the absence of trypsin. (A) Replication kinetics of HPAI H5N1^WT^ and H5N1^ΔMBCS^ viruses in SK-N-SH, U87-MG, and MDCK cells (MOI of 0.1) in the presence or absence of trypsin. Statistical analysis was performed using a two-way ANOVA with Tukey’s multiple-comparison test against “H5N1WT + trypsin.” (B) Percentages of infection in SK-N-SH, U87-MG, and MDCK cells were determined by FACS at 8 hpi with HPAI H5N1^WT^ and H5N1^ΔMBCS^ viruses (MOI of 3). Statistical analysis was performed using the two-way ANOVA with Bonferroni’s multiple-comparison test. (C) Percentages of infection in SK-N-SH, U87-MG, and MDCK cells were determined by FACS at 8 and 24 hpi with HPAI H5N1^WT^ and H5N1^ΔMBCS^ viruses at an MOI of 0.1 in the absence of trypsin. Statistical analysis was performed using the two-way ANOVA with Bonferroni’s multiple-comparison test (8 versus 24 hpi). All data are presented as means ± SDs from at least three independent experiments. *, *P* ≤ 0.05; **, *P* ≤ 0.01; ***, *P* ≤ 0.001; ****, *P* ≤ 0.0001.

To determine whether efficient replication of HPAI H5N1 virus in cell culture solely depends on the presence of the MBCS, we generated an H5N1 virus without the MBCS. The replication kinetics of the HPAI H5N1^WT^ (wild-type) virus was not affected by the presence or absence of trypsin in all cell lines **(**Fig. 6A). However, the H5N1^ΔMBCS^ virus, without trypsin, replicated to a lower titer on each cell line investigated. This phenotype was restored by the addition of trypsin to the culture medium, allowing the virus to replicate to wild-type levels (Fig. 6A). The reduced replication efficiency of H5N1^ΔMBCS^ virus was not explained by the ability of the virus to infect cells, since this was not affected (Fig. 6B). In order to determine if there were multiple rounds of infection and that virus detected in the supernatant was not solely the result of primary infected cells, we investigated the percentage of infection at 8 and 24 hpi with an MOI of 0.1, without trypsin, measured by flow cytometry. We found that HPAI H5N1^WT^ virus efficiently replicated in MDCK, SK-N-SH, and U87-MG cells as indicated by the increase of infection percentages (Fig. 6C). In contrast, a significant increase for H5N1^ΔMBCS^ virus only was observed in MDCK and U87-MG cells. In SK-N-SH cells, no increased infection percentage was observed. These results reveal that the MBCS is important but not solely responsible for efficient replication in MDCK and U87-MG cells in the absence of trypsin.

## DISCUSSION

Here we show that HPAI H5N1 virus replicates more efficiently in human and mouse neuronal cells than seasonal H3N2 and 2009 pandemic H1N1 viruses. Both the ability to attach efficiently and the presence of an MBCS of HPAI H5N1 virus contributed to efficient replication in cells of the CNS, indicative that these are viral factors that contribute to the neurotropic potential of influenza viruses. This fits with the facts that HPAI H5N1 virus is more frequently associated with CNS disease in humans than seasonal and pandemic viruses (1) and that this virus is also more often detected in tissues of the CNS in experimentally inoculated laboratory animals than seasonal and pandemic viruses (17, 20–27, 51, 55–58).

The ability of HPAI H5N1 and WSN viruses to replicate efficiently in cells of the CNS seems to be associated with the ability to attach to and infect host cells efficiently. Especially, HPAI H5N1 virus which replicated efficiently in SK-N-SH and U87-MG cells, attached to high percentages of cells with high intensity, and infected these cells efficiently. WSN virus attached to and infected high percentages of neuronal cells, which resulted in multiple rounds of infection in all cells, although to lower titers on SK-N-SH and U87-MG cells than observed for HPAI H5N1 virus. The latter could be due to the fact that WSN virus is extensively passaged in suckling mouse brains, thereby adapting to mouse neuronal cells and not human neuronal cells. Seasonal H3N2 viruses replicated in SK-N-SH cells, even in the absence of efficient attachment or infection. Whether this is due to low-affinity binding of H3N2 virus, which cannot be detected by our assays, is unknown, but it does suggest that efficient attachment, as observed for H5N1 and WSN viruses on neuronal cells, is not the only viral factor involved in replication of influenza viruses in cells of the CNS. The lack of replication of H3N2 and pH1N1 viruses in U87-MG cells could be explained by both inefficient attachment and infection.

Efficient replication of influenza viruses in cells of the CNS seems to depend in part on the presence of an MBCS or alternative HA cleaving mechanisms. Studies in ferrets, mice, macaques, and chickens show that introduction or removal of an MBCS has different outcomes based on the virus backbone and host species (24, 48, 59–61). *In vitro*, in the absence of trypsin, only HPAI H5N1 and WSN viruses were able to replicate, indicating that pH1N1 and H3N2 viruses are not able to circumvent the need for trypsin-like protease for HA cleavage. Removal of the MBCS from HPAI H5N1 virus resulted in attenuated replication in SK-N-SH and U87-MG cells in the absence of trypsin but not in pmCortex cells. Viruses without an MBCS that are associated with replication in cells of the CNS *in vivo*, such as the 1918 H1N1 and WSN viruses, have an alternative HA cleavage mechanism (6, 62). The WSN virus lacks a conserved glycosylation site in the neuraminidase making the virus trypsin independent (63) by using the serine-protease plasmin, which is present in many organ systems besides the respiratory tract (64). The 1918 H1N1 virus grows trypsin independent and neuraminidase dependent in MDCK cells and polarized Calu-3 cells but not in Huh-7 cells (65, 66). Our observation that neither HAT, TMPRSS2, nor TMPRSS4 is found in SK-N-SH and U87-MG cells supports the hypothesis that for efficient replication in cells of the CNS, influenza viruses require alternative HA cleavage, as shown for the HPAI H5N1, WSN, and 1918 H1N1 viruses. However, it must be noted that there is limited knowledge on the expression and accessibility of proteases in tissues other than the respiratory tract.

Previous studies on the replication kinetics of influenza viruses in cells of the CNS revealed some differences, which can in part be explained by the use of different cells, virus isolates, and experimental approaches (36, 54). Replication of HPAI H5N1 viruses in differentiated astrocytic cell lines resulted in efficient replication, similar to our observations. However, in SH-SY5Y cells, a subclone cell line derived from SK-N-SH cells, two HPAI H5N1 viruses did not replicate efficiently. This discrepancy could be due to the relatively high MOI used in this study compared to the low MOI we used and which resulted in efficient replication (36). Furthermore, two pandemic H1N1 viruses did not replicate in the neuronal or astrocytic cell lines, which fits with our observation, in the absence of trypsin (54).

Pandemic H1N1 and seasonal H3N2 viruses are occasionally detected in the CNS or CSF in humans and from experimentally inoculated ferrets and mice, even though pH1N1 and H3N2 viruses only replicated in SK-N-SH cells in the presence of trypsin (11–15, 67–70). However, these viruses are rarely isolated in high titers or detected by immunohistochemistry in the CNS of humans or experimentally inoculated ferrets and mice, indicating that these viruses might be able to enter the CNS but that replication is inefficient. This could be attributed to the limited attachment and infection and lack of an alternative HA cleavage mechanism allowing efficient replication. Previously, we showed that even in the absence of active virus replication, proinflammatory cytokines, such as interleukin 6 (IL-6), IL-8, and tumor necrosis factor alpha (TNF-α), are induced in the CNS of pH1N1 experimentally inoculated ferrets (71). Future studies should reveal how both efficient and inefficient replication in neuronal cells can trigger local proinflammatory responses, for which HPAI H5N1 and H5N1^ΔMBCS^ viruses might be a good model.

Taken together, results of our study have shown that the presence of an MBCS and, to a lesser extent, the ability to attach are important determinants for replication of HPAI H5N1 virus in cells of the CNS. This suggests that, at least for replication within the CNS, neurotropic influenza viruses contain an alternative HA cleavage mechanism and prefer α-2,3-linked sialic acids.

## MATERIALS AND METHODS

### Cells.

Human neuroblastoma (neuron like, SK-N-SH) and human glioblastoma (astrocyte like, U87-MG) cells were purchased from Sigma-Aldrich and maintained in Eagle minimal essential medium (EMEM; Lonza, Breda, the Netherlands) supplemented with 10% fetal bovine serum (FBS; Sigma-Aldrich, St. Louis, MO, USA), 100 IU/ml penicillin (Lonza, Basel, Switzerland), 100 μg/ml streptomycin (Lonza), 2 mM glutamine (Lonza), 1.5 mg/ml sodium bicarbonate (Cambrex, Wiesbaden, Germany), sodium pyruvate (Thermo Fisher Scientific, Waltham, MA, USA) and 1× (0.1 mM) nonessential amino acids(MP Biomedicals Europe, Illkirch, France). As a control cell line, we have included Madin-Darby canine kidney (MDCK) cells, since these cells are extensively used for influenza virus propagation. MDCK cells were maintained in EMEM supplemented with 10% FBS, 100 IU/ml penicillin, 100 μg/ml streptomycin, 2 mM glutamine, 1.5 mg/ml sodium bicarbonate, 1 mM, 10 mM HEPES (Cambrex), and 1× (0.1 mM) nonessential amino acids.

### Viruses.

Five viruses were included in this study, a seasonal H3N2 virus (A/Netherlands/213/2003), pH1N1 virus (A/Netherlands/602/2009), and zoonotic HPAI H5N1 virus (A/Indonesia/5/2005) all isolated from humans. Neurotropic WSN virus (A/WSN/33) and H5N1 virus lacking an MBCS (H5N1^ΔMBCS^) were generated using reverse genetics as described before (72) and passed once on 293T cells and once on MDCK cells. Experiments involving HPAI H5N1 and H5N1^ΔMBCS^ viruses were performed under biosafety level 3 conditions.

### Isolation and culture of primary mouse cortex neurons.

Animals were housed and experiments were conducted in strict compliance with European guidelines (EU directive on animal testing 86/609/EEC) and Dutch legislation (Experiments on Animals Act, 1997). Primary mouse cortex tissue was isolated from embryonic day 17 (E17) to E19 C57BL6 mouse embryos (Charles River Laboratories, Wilmington, MA, USA). The cultures were pooled cortexes of several mouse embryos originating from one mother. In brief, the cortex was dissected in ice-cold Hanks’ balanced salt solution (HBSS; Life Technologies) supplemented with 20 μg/ml gentamicin (Life Technologies) under guidance with a stereomicroscope (Nikon). Next, tissues were cut to ∼1 mm^3^ using a scalpel and digested using medium consisting of HBSS supplemented with 10 U/ml papain (Sigma), 2.5 U/ml DNase I (Roche), and 4 mM MgCl_2_ (Sigma-Aldrich) at 33°C for 15 min. After incubation, cells were washed once in 1 ml of 10% FBS (Life Technologies) in HBSS to stop the digestion. A second “mechanical digestion” was performed by carefully pipetting up and down in digestion buffer (without papain). After washing in HBSS twice, cells were counted using a Moxi Go cell counter (Orflo, Ketchum, ID, USA) and seeded on laminin (500 μg/ml; Sigma)-coated 1.5H 96-well glass-bottomed plates (Cellvis, Sunnyvale, CA, USA) at a density of 1.0 × 10^4^ cells/well. For the first 2 h, the cells were cultured in culture medium containing 10% FBS. After 2 h, medium was replaced with fresh culture medium, without FBS. The culture medium contains primary neuron growth medium (PNBM; Lonza), GS-21 supplement (Tebu-Bio, Le-Perray-en-Yvelines, France), 5 μg/ml gentamicin (Thermo Fisher), and 2 mM GlutaMAX (Life Technologies). Half of the medium was changed once per week, and cells were cultured for 7 to 10 days before use.

### Replication kinetics.

Cells were infected at a multiplicity of infection (MOI) of 0.1. Virus dilutions were prepared in the cell-specific culture medium without serum (infection medium, see “Virus titrations”). After 1 h of virus absorption, cells were washed once with fresh infection medium and cultured in infection medium in the presence or absence of l-1-tosylamide-2-phenylethyl chloromethyl ketone (TPCK)-treated trypsin (see “Virus titration”). At time points 1, 6, 24, and 48 h postinfection (hpi), 100 μl supernatant was collected and stored at −80°C for subsequent virus titration. All experiments were performed three times (biological replicates), and each experiment was performed with duplicates (technical replicates) from which the average was used for statistical analysis.

### Virus titrations.

The 50% tissue culture infectious dose (TCID_50_) in cell supernatant was determined by endpoint titration on MDCK cells, as described before (73). Briefly, 10-fold serial dilutions of cell supernatants were prepared in infection medium. Infection medium consisted of EMEM, 100 IU/ml penicillin, 100 μg/ml streptomycin, 2 mM glutamine, 1.5 mg/ml sodium bicarbonate, 10 mM HEPES, 1× (0.1 mM) nonessential amino acids, and 1 μg/μl TPCK-treated trypsin (Sigma-Aldrich). Before inoculation, MDCK cells were washed twice with phosphate-buffered saline (PBS) to remove remaining FBS. One hundred microliters of the diluted supernatant was used to inoculate a confluent monolayer of MDCK cells in 96-well plates. After 1 h at 37°C, the cells were washed once with infection medium, and 200 μl new infection medium was added to each well. Three days after infection, supernatants of infected cell cultures were tested for agglutinating activity using turkey erythrocytes as an indicator of virus replication. The titers of infectivity were calculated from three replicates according to the method of Kärber (74). An initial 1:10 dilution of supernatant resulted in a detection limit of 10^1.5^ TCID_50_/ml.

### Percentage of infection.

After 8 hpi (with an MOI of 3) or after 8 and 24 hpi (with an MOI of 0.1), in the absence of trypsin, cells were collected, fixed, and permeabilized using BD Cytofix/Cytoperm solution (BD Biosciences, Franklin Lakes, NJ, USA) according to the manufacturer’s instructions. Cells were incubated with 2% normal goat serum (NGS; Dako, Denmark) in PBS for 10 min on ice. Next, influenza A virus was detected using a monoclonal antibody against influenza A virus nucleoprotein (clone HB-65, 1 μg/ml; ATCC) or mouse IgG2a isotype control (MAB003, 1 μg/ml; Dako) in BD Perm/Wash containing 2% NGS and incubated for 1 h on ice and in the dark. Cells were washed twice and incubated with goat anti-mouse IgG2a conjugated to Alexa Fluor 488 (8 μg/ml; Life Technologies, Inc., the Netherlands) for 1 h in the dark and on ice. After incubation, cells were washed twice and resuspended in fluorescence-activated cell sorting (FACS) buffer. Cells were measured and data collected using a BD FACSCanto II (BD Biosciences, USA). Data were analyzed using FlowJo 10 software (Ashland, OR, USA). All experiments were performed three times (biological replicates), and each experiment included duplicate (technical replicate) measurements from which the average was calculated and used for further analysis.

### Virus attachment.

For influenza virus histochemistry, viruses were grown, inactivated, and labeled as described previously (32). As a control, uninfected MDCK cells and cell debris were harvested and processed similarly. Subsequently, in a 12-well plate, 2 × 10^5^ cells were seeded, and 1 day later, the near confluent monolayers of MDCK, SK-N-SH, and U87-MG cells were harvested, washed in FACS buffer, and incubated with fluorescein isothiocyanate (FITC)-labeled virus for 1 h at 4°C. After incubation, the cells were washed twice in FACS buffer and measured using a BD FACSCanto II (BD Biosciences, USA). Data were analyzed using FlowJo 10 software (Ashland, OR, USA). All experiments were performed three times (biological replicates), and each experiment was performed with duplicates (technical replicates) from which the average was used for statistical analysis.

### PCR proteases.

Since MDCK cells are of canine origin, we have included primary human nasal (HN) cells (MucilAir, pool of 14 donors; Epithelix, Geneva, Switzerland), and primary human bronchial/tracheal epithelial cells (HBTE) (catalog number CC-2540, lot 97366, donor 97366: male, Caucasian, 57 years, healthy; Lonza) as control cell types for the expression of human HAT, human TMPRSS2, and human TMPRSS4. Total RNA was isolated from SK-N-SH, U87-MG, HN, and HBTE cells using the High Pure RNA isolation kit (Roche, Basel, Switzerland) according to the manufacturer’s protocol. cDNA synthesis was performed using oligo(dT) primers and Superscript IV (Applied Biosystems, Foster City, CA, USA) according to the manufacturer’s instructions. For detection of HAT-, TMPRSS2-, and TMPRSS4-specific mRNAs, primers were used from Böttcher-Friebertshäuser et al. (75). The GAPDH (glyceraldehyde-3-phosphate dehydrogenase) mRNA was detected using primers GAPDH-FW (5′-TGA ACG GGA AGC TCA CTG G-3′) and GAPDH-RV (5′-TCC ACC ACC CTG TTG CTG TA-3′) as a control for sample quality. PCR products were resolved on a 1.5% agarose gel stained with SYBR Safe (Thermo Fisher) and imaged using a ChemiDoc MP imaging system and ImageLab 5.1 (Bio-Rad, Hercules, CA, USA). To confirm the specificity of the primers, PCR products were extracted from the gel and sequenced using a BigDye Terminator v3.1 Cycle sequencing kit (Applied Biosystems) and a 3130XL genetic analyzer (Applied Biosystems), according to the instructions of the manufacturer.

### Statistical analysis.

Statistical analyses were performed using GraphPad Prism 6.0h software (La Jolla, CA, USA) for Mac. Each specific test is indicated in the figure legends. *P* values of ≤0.05 were considered significant. All data are presented as means ± standard deviations (SDs) from at least three independent experiments.
